# Arterial Stiffness and Wave Reflection Responses Following Heavy and Moderate Load Resistance Training Protocols

**DOI:** 10.1111/jch.70020

**Published:** 2025-04-10

**Authors:** Eleftherios Karanasios, Scott Hannah, Helen Ryan ‐ Stewart, James Faulkner

**Affiliations:** ^1^ School of Sport Health and Community Faculty of Health & Wellbeing University of Winchester Winchester UK; ^2^ School of Health and Sport Science Faculty of Education Humanities and Health Science Eastern Institute of Technology Taradale New Zealand

**Keywords:** arterial stiffness, intensity of effort, loading intensity, resistance training

## Abstract

This study compared the acute effects of resistance training (RT) between a moderate (ML) and a high loading (HL) intensity (12RM vs. 4RM, respectively), with the same intensity of effort on arterial stiffness and wave reflection in young healthy adults. Eleven healthy adults (age 36.4 ± 6.8 years) performed two RT protocols, ML and HL, in a randomized order. Both RT sessions consisted of three sets of deadlifts and three sets of bench presses, with 2 min rest between sets and exercises. Loading intensity was 12RM and 4RM for the ML and HL conditions, respectively. Measurements of pulse wave velocity (PWV) and pulse wave analysis (PWA; e.g., augmentation index) were collected at baseline, immediately post, and 15 min post‐training. ML elicited significantly greater increases in carotid‐femoral PWV (from 6.4 ± 0.3 to 7.3 ± 0.5), and augmentation index normalized to 75 bpm (from −5.1 ± 1.1) than HL (all *p* < 0.05). These findings demonstrate that an acute bout of RT performed to volitional failure using lower loads and higher repetitions impose a greater workload on the arterial and cardiovascular system in comparison to a RT scheme with heavier loads and lower repetitions.

## 1 Introduction

Arterial stiffness (AS) is an independent predictor of cardiovascular disease (CVD) and is strongly associated with hypertension and future cardiovascular events [1]. Regular participation in resistance training (RT) has been endorsed by current guidelines as a promising intervention for the prevention and treatment of CVD [2]. Yet, the effects of RT on AS are not fully understood and remain to be ascertained [3]. Interestingly, increases [4], decreases [5], and no changes [6] in AS have been reported following both acute and chronic RT interventions. Differences in the loading characteristics among the prescribed RT protocols (i.e., loading intensities, proximity to failure) may account for the discrepancies observed [3].

Loading intensity is a well‐researched training variable particularly in the field of muscular hypertrophy and strength [7], yet there is a paucity of research directly examining the effects of different loading intensities on AS. One of few studies to directly investigate loading and AS compared the effects of heavier (75%–90% 1‐repetition maximum [1RM]) versus lighter load (30%–50% 1RM) RT on AS and reported a reduction in AS regardless of the load lifted [9]. Elsewhere, Werner et al. [6] reported no differences in AS after a 12‐week RT program performed using high (80%–90% 1RM) or moderate (50%–70% 1RM) loading intensities. Nitzsche et al. [18] reported acute increases in AS following low (30% 1RM) and moderate (50% 1RM) intensities but not following a higher loading intensity (70% 1RM) in young healthy adults. These findings contrast a recent meta‐analysis which suggested that loading intensity is the key variable determining arterial responses to RT [8]. It was noted that low to moderate loading intensities (<70% 1RM) tend to decrease AS, whereas high loading intensities (>70% 1RM) seem to increase AS particularly in young individuals [8]. Nonetheless, the lack of control over proximity to failure in previous reports may be a confounding factor when interpreting these findings [6, 9].

Proximity to failure operationalized as “intensity of effort” [10], appears to be a major determinant of cardiovascular [11] and metabolic [12] responses to RT. Notably, loading intensity in the study by Nitzsche et al. [18] was prescribed in relative terms (i.e., as a percentage of 1RM) with a fixed number of repetitions (i.e., three sets of 10 repetitions at 70% of 1RM), despite previous evidence suggesting large variability in the number of repetitions that can be performed at given relative load by different individuals [13]. That said, it has recently been suggested that standardization of effort allows for a more appropriate comparison of other training variables, such as load, since it ensures a more uniform stimulus and establishes parity among the conditions or groups under investigation [14].

High and low loading intensities may elicit similar hypertrophic responses [7]; however, high loads may induce superior neural adaptations [15]. Notably, muscular strength exhibits a stronger inverse relationship with AS than muscle mass alone across a wide age (20–75 years) span [16]. Thus, identifying the potential impact of loading intensity on AS would assist in determining the most effective RT prescription for improved vascular adaptations.

To date, there is no study that has directly compared the effects of load on AS while standardizing proximity to failure. Thus, the purpose of this study was to compare indices of AS between a strength‐type RT scheme using heavier loads (4RM) and lower repetitions with a hypertrophy‐type RT protocol using moderate loads and repetitions (12RM) while maintaining intensity of effort constant. Based on the available evidence, it was hypothesized that the high load session would increase AS, whereas the moderate load would reduce or not significantly affect AS.

## 2 Methods

### 2.1 Participants

Eleven young healthy participants (five males, six females) volunteered to participate in this study. Participants were classified as recreationally active who had been participating in RT (e.g., with free weights or weight machines) at least once a week for the last 6 months. All participants were in a good state of health, non‐hypertensive, and free of any cardiovascular, musculoskeletal, and metabolic disease. All female participants voluntarily reported that they were tested during the follicular phase of their menstrual cycle, although previous research indicates minimal influence of different menstrual cycles on AS measured by cfPWV [17]. Participants were not taking any prescribed medications known to affect vascular function during the study period. Prior to participating in the study and after being informed of the study's procedures, all participants provided written informed consent. The study was approved by the Faculty of Health and Wellbeing Research Ethics Committee at the University of Winchester.

### 2.2 Sample Size Calculation

Sample size was calculated using G*Power 3.1 (version 3.1.9.7, Heine University, Düsseldorf, Germany). The effect size was estimated based upon a between‐group (e.g., heavier vs. lighter load) partial eta squared ($\eta_{p}^{2})$ effect size of 0.114 reported for acute cfPWV in a previous study [18]. Thus, for repeated measures within factors design, with an effect size *F* = 0.35, a significance level of 0.05, a power of 0.80, and a correlation among repeated measurements of 0.70 [19], a sample size of 11 participants was found to be sufficient to show a difference between higher and lighter load RT conditions, while accounting for a 10% drop‐out.

### 2.3 Study Design

**FIGURE 1:**
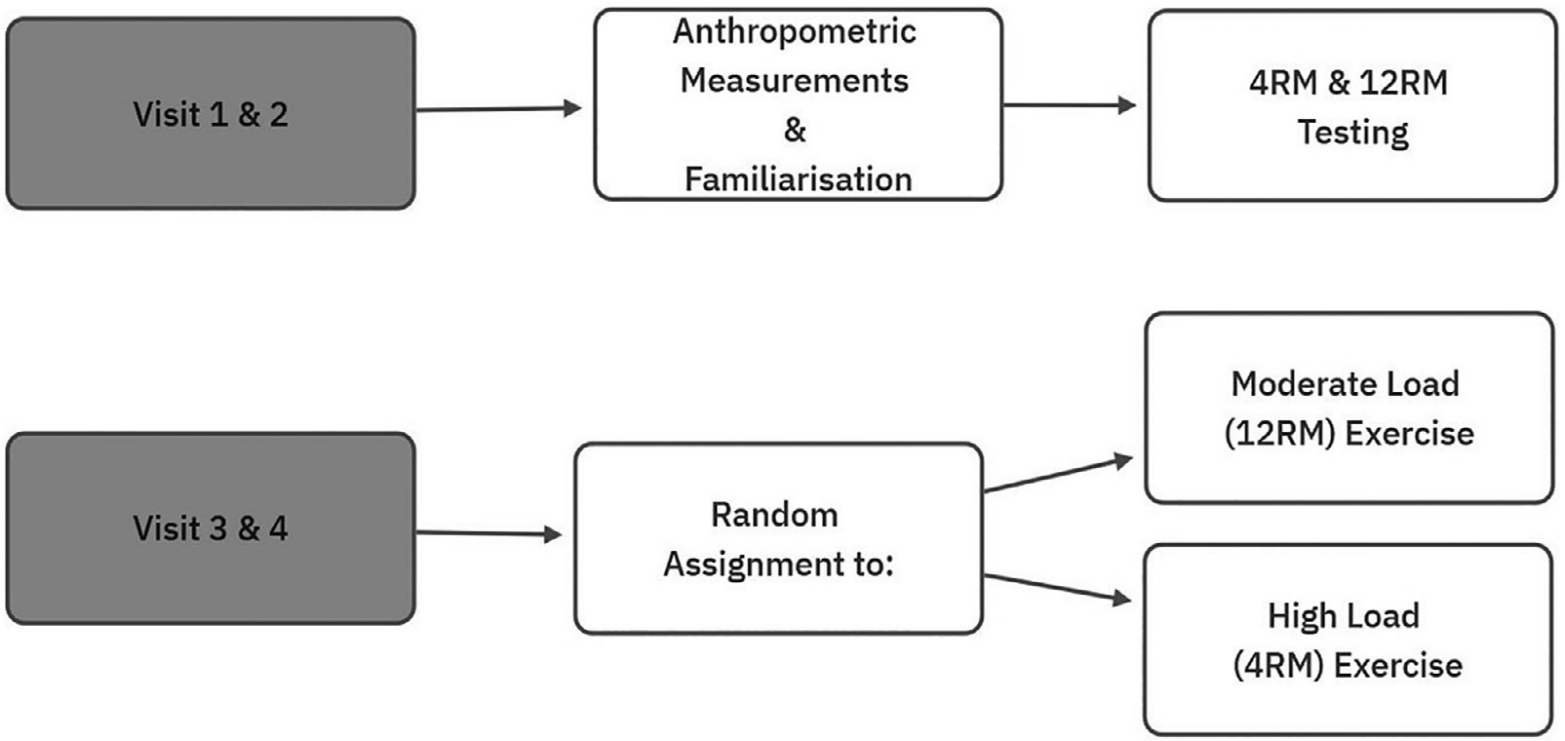
Flowchart illustrating the research process.

Participants took part in a randomized cross‐over design and reported to the research facility on four separate occasions. The first two visits consisted of anthropometric measurements (i.e., height and body mass), and a familiarization session in which participants were introduced to the experimental procedures and RM testing, to determine individual 4RM and 12RM training loads for the deadlift and bench press exercise. Loading prescription of the two RT sessions was based upon the “repetition continuum” also known as the “strength‐endurance continuum” [20]. According to the repetition continuum strength gains are maximized by a low repetition strategy with heavy loads (1–5 repetitions per set with 80%–100% of 1RM) while a moderate repetition scheme with moderate loads (e.g., 8–12 repetitions per set at 60%–80% of 1RM) maximizes hypertrophic adaptations [21]. In the third and fourth visits, participants were randomly assigned RT protocol using either a 4RM (High Load; HL) or 12RM (Moderate Load; ML). Participants refrained from strenuous physical activity for 24 h and large meals and caffeine consumption for at least 4 h prior to testing [22]. The research process is illustrated in Figure 1.

### 2.4 RM Testing Protocol and Experimental Sessions

RM testing protocol (visits 1 and 2)

The maximum amount of weight lifted for 12 and 4 repetitions with proper form in the deadlift and bench press exercise was recorded as the participant's 12RM and 4RM load, respectively. This was determined following the guidelines proposed by the National Strength and Conditioning Association [23]. All RM loads were determined in no more than five attempts with rest periods of 3 min between the trials.

Experimental sessions (visits 3 and 4)

Two RT sessions comprised the experimental protocol, one for each condition (HL and ML). Both RT sessions consisted of three sets of deadlifts and three sets of bench press. Rest duration was 2 min between sets, and 2 min between exercises, for both conditions. Participants were guided by the researcher to adopt a repetition tempo of approximately 1 s concentric, 2 s eccentric, and 1 s isometric pause between concentric and eccentric actions. Intensity of effort was standardized between the experimental conditions and during both sessions exercises were performed to volitional failure (i.e., the exercise was terminated once trainees determined they could not complete further repetitions if attempted [24]). If a participant reached momentary failure (i.e., the inability to perform another concentric repetition despite attempting to [25]) before performing the expected number of repetitions (i.e., 12 or 4) in a single set, the set was terminated without any further attempts to complete the required number of repetitions. Loading intensity was set at 12RM and 4RM for the ML and HL condition, respectively.

### 2.5 Hemodynamic Measurements

Hemodynamic measurements were collected three times during the experimental procedure: before training (baseline), immediately post (Post), and 15 min post‐training (15Post). All measurements were collected in a supine position. The first hemodynamic data collection was conducted after 10 min of rest in a supine position [22]). Following that, participants completed the warm‐up routine as described above, and then completed their randomly assigned RT protocol. Participants returned to the supine position immediately post the acute RT protocol while data collection equipment was applied and remained supine until completion of 15Post assessments.

All hemodynamic measurements were performed using a validated oscillometric cuff‐based device (Vicorder, Skidmore medical, Bristol, UK), that calculates pulse wave velocity (PWV) by simultaneously recording the upstroke of the femoral and carotid pulsations [26]. Two inflatable cuffs were used to measure Carotid‐femoral PWV (cfPWV), one placed around the neck over the carotid artery and the other around the thigh over the femoral artery. CfPWV was calculated by dividing the pulse wave travel distance by the pulse transit time between the two recording sites. Transit time was determined by the software using an in‐built cross‐correlation algorithm and travel distance length was defined as the distance from the suprasternal notch to the mid‐upper thigh cuff, as indicated by the manufacturer. Augmentation index is an indicator of AS which reflects the augmentation of systolic blood pressure by reflection of the peripheral pulse wave. AIx was calculated as the ratio between the augmentation pressure (AP) and the central pulse pressure (Cpp) and was expressed as a percentage (AIx = AP/Cpp × 100). AIx was normalized to 75 beats per minute (bpm) to reduce its reliance on HR(AIx@75), as previously suggested [27]. Measurements of subendocardial viability ratio (SEVR), an index representing myocardial perfusion, which is calculated as the ratio between diastolic pressure time index (DPTI) and systolic pressure time index (SPTI) [28]; and of central and peripheral blood pressures were performed using a cuff placed around the upper arm over the brachial artery and assessed using device‐specific pulse wave analysis [29].

### 2.6 Statistical Analysis

All descriptive data are expressed as means ± standard deviation. Normality of distribution was assessed by the Shapiro–Wilk test and visual inspection. A two‐way (Condition × Time) repeated measured analysis of variance (ANOVA) was conducted to evaluate the effects of condition, time, and the interaction between them for: cfPWV; Aix; AIx@75; central systolic blood pressure (cSBP); central diastolic blood pressure (cDBP); peripheral systolic (pSBP); peripheral diastolic (pDBP); AP; mean central arterial pressure (cMAP); and SEVR. Violations of sphericity were adjusted for using the Greenhouse–Geisser correction. Post hoc pairwise comparisons with a Bonferroni correction were conducted when Condition by Time interactions were detected. Analysis of the effect size was conducted using the partial Eta squared ($\eta_{p}^{2}$). The magnitude of effect size was interpreted as trivial (<0.01), small (0.01–0.06), moderate (>0.06–0.14), and large (>0.14) [30]. Significance was set at *p* < 0.05. Data were analyzed using SPSS version 28 statistical software (SPSS Inc, Chicago, IL, USA). A linear mixed‐effects model (Jamovi, version 2.3.28) was also employed to evaluate the effect of the intervention on cfPWV (primary outcome), controlling forcMAP as a covariate [22]. Random intercepts were included for each subject to account for repeated measurements over time. Fixed effects in the model included Condition, Time, and the interaction between Condition and Time, while cMAP was included as a covariate to account for its effect on cfPWV. Residual plots were examined to verify model assumptions of normality and homoscedasticity.

## 3 Results

Characteristics of the study participants are displayed in Table 1.

**TABLE 1:** Participant characteristics.

		ML (12RM)	HL (4RM)
Age (year)	36.4 ± 6.8		
Weight (kg)	67.3 ± 12.0		
Height (cm)	172.8 ± 7.8		
Dead lift (kg)		56.6 ± 15.0	76.8 ± 16.6
Bench press (kg)		40.6 ± 10.9	52.4 ± 15.8
Volume (repetitions)		71.2 ± 0.8	23.8 ± 0.4
Volume load (kg)		3419.6 ± 884.8	1540.6 ± 384.4

*Notes*: Volume load is calculated as: sets × repetitions × load. Data presented are mean ± SD.

Abbreviations: BP, bench press; DL, deadlift; HL, high load; ML, moderate load.

There were no significant differences between the experimental conditions at baseline (BL) for any of the variables analyzed (all *p* > 0.05).

**FIGURE 2:**
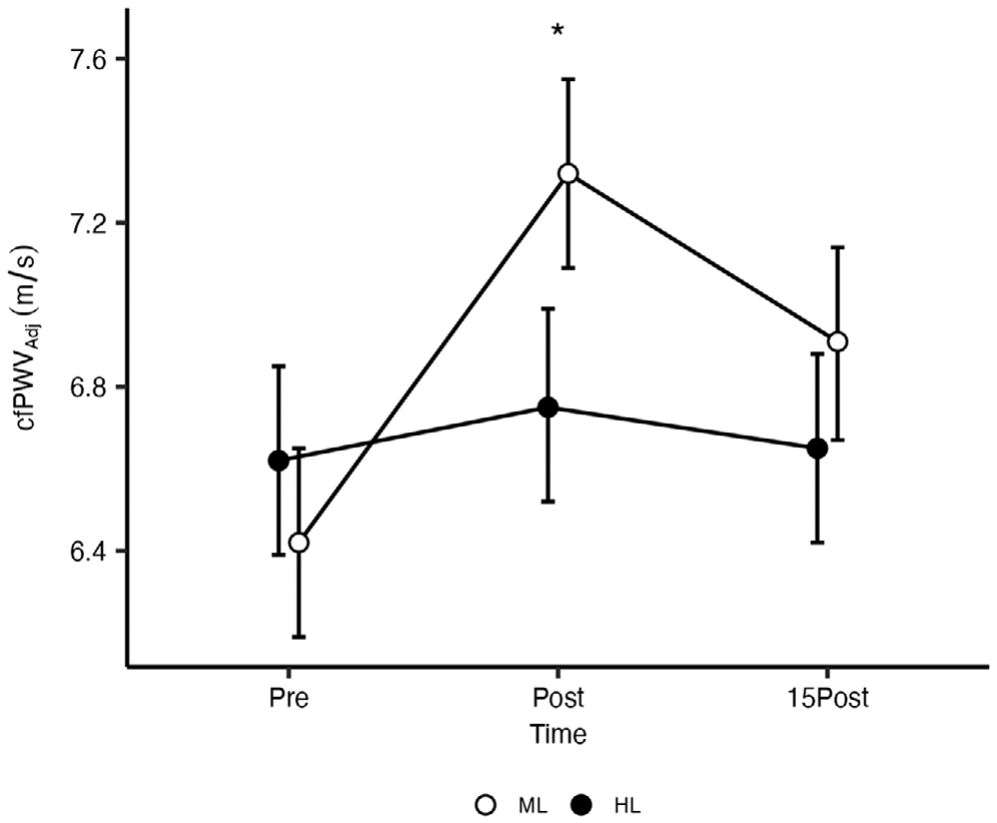
Changes in cfPWV measured at rest and following the RT protocols. cfPWV Adj, carotid‐femoral pulse wave velocity adjusted for cMAP; ML, moderate load; HL, high load; Interaction effect at the post exercise timepoint (*p* < 0.001). Data are presented as means and confidence intervals 95%.

Hemodynamic data are shown in Table 2. Significant Condition and Time interactions were observed for cfPWV ($\eta_{p}^{2}=0.47$), AIx ($\eta_{p}^{2}=0.4$), and AP ($\eta_{p}^{2}=0.42)$ (all *p* < 0.05). Post hoc analysis revealed that changes from BL to Post for cfPWV, AIx, and AP were significantly higher in the ML compared to HL. In the ML condition, cfPWV, was significantly greater than BL at both Post and 15Post, but significantly decreased from Post to 15Post (*p* < 0.05). AIx and AP showed a similar trend and were significantly greater than BL at both Post and 15Post following the ML condition (all *p* < 0.05). In the HL Condition no significant differences in cfPWV from BL were observed, while AIx and AP significantly increased between BL and Post only. cMAP had no significant main effect on cfPWV (*p* = 0.14), therefore a similar statistical Condition by Time interaction was observed for cfPWV when controlling for cMAP (*p* < 0.001) (Figure 2). There were no significant Condition by Time interactions for all other variables (Table 2).

**TABLE 2:** Hemodynamic and cardiovascular variables at rest and during recovery from an acute ML (12RM) and HL (4RM) resistance training protocol.

	Time point			Interaction effect	
	Baseline	Post	15Post	*p* for interaction effect	Effect size (η^2^_p_)
**cSBP (mm Hg)** ^b^					
ML	111.4 ± 11.0	119.9 ± 10.2	111.3 ± 14	0.239	0.13
HL	112.7 ± 10.1	117.6 ± 14.9	115.7 ± 15.6		
Total	112 ± 9.9	118.7 ± 9.9	113.5 ± 13.6		
**cDBP (mm Hg)** ^b^					
ML	60.5 ± 8.5	47.8 ± 6.6	50.2 ± 11.0	0.180	0.15
HL	60.3 ± 8.6	56.6 ± 12.5	56.8 ± 11.7		
Total	60.3 ± 8	52.2 ± 7.3	53.5 ± 8.3		
**cfPWV (m/s)**					
ML	6.4 ± 0.3	7.3 ± 0.5	6.9 ± 0.4	0.002	0.47
HL	6.6 ± 0.5	6.8 ± 0.3	6.6 ± 0.2		
Total	6.5 ± 0.3	7 ± 0.3	6.7 ± 0.3		
**cfPWV‐adjusted (m/s)**					
ML	6.4 ± 0.3	7.3 ± 0.3	6.9 ± 0.3		
HL	6.6 ± 0.3	6.7 ± 0.3	6.6 ± 0.3	<0.001	
Total	6.5 ± 0.3	7 ± 0.3	6.7 ± 0.3		
**AIx (%)**					
ML	12.5 ± 5.4	32.5 ± 10.7	17.2 ± 6.2	0.018	0.40
HL	14.3 ± 7.3	23 ± 7.9	14.8 ± 8.9		
Total	13.3 ± 5.6	27.7 ± 7.6	16 ± 7		
**AIx@75 (%)** ^a^, ^b^					
ML	−5.1 ± 3.3	0.6 ± 7.7	−2.0 ± 6.1	0.210	0.14
HL	−5.7 ± 4.3	−3.0 ± 5.9	−4.3 ± 4.7		
Total	−5.4 ± 3.6	−1.1 ± 6	−3.1 ± 5		
**AP (mm Hg)**					
ML	6.5 ± 3.2	23.5 ± 9.6	11 ± 7.3	0.004	0.42
HL	7.5 ± 3.9	14.2 ± 6.2	9.5 ± 7.5		
Total	6.9 ± 3	18.8 ± 6.3	10.2 ± 6.3		
**pSBP (mm Hg)**					
ML	118.4 ± 8.8	123.2 ± 10.1	116.4 ± 13	0.131	0.18
HL	119.5 ± 8.9	121.3 ± 14.1	122.8 ± 12.7		
Total	118.9 ± 8	122.2 ± 9.3	119.5 ± 11.3		
**pDBP (mm Hg)** ^b^					
ML	60.5 ± 8.5	47.8 ± 6.6	50.2 ± 11	0.130	0.18
HL	59.8 ± 8.8	57.4 ± 13.2	56.8 ± 11.7		
Total	60.1 ± 8.3	52.5 ± 7.6	53.5 ± 8.3		
**HR (bpm)** ^a^, ^b^					
ML	64.1 ± 6.8	75.5 ± 16.0	70.5 ± 12.6	0.236	0.13
HL	62.8 ± 9.0	68.4 ± 12.2	65.8 ± 9.7		
Total	63.4 ± 7.3	71.9 ± 12.9	68.1 ± 10.6		
**MAP (mm Hg)**					
ML	83 ± 10.8	82.4 ± 8.0	79.6 ± 10.3	0.782	0.02
HL	83.5 ± 9.7	85.1 ± 13.1	82.5 ± 12.2		
Total	83.2 ± 9.9	83.7 ± 8.6	81.1 ± 10.3		
**SEVR (%)** ^a^, ^b^					
ML	160.6 ± 18.8	128.5 ± 26.9	131.1 ± 36.2	0.058	0.24
HL	158.3 ± 18.9	146.8 ± 27.5	138.8 ± 20.2		
Total	159.4 ± 17.2	137.6 ± 26.5	134.9 ± 26.2		

*Notes*: Data are displayed as means ± SD; ¥ interaction main effect (*p* < 0.05).

Abbreviations: AIx, augmentation index; Aix@75, augmentation index normalised at 75bmp; AP, augmentation pressure; cDBP, central diastolic blood pressure; cfPWV, carotid‐femoral pulse wave velocity; cfPWV‐adjusted, cfPWV adjusted for mean arterial pressure; cMAP, central mean arterial pressure; cSBP, central systolic blood pressure; HL, high load; HR, heart rate; ML, moderate load; pDBP, peripheral diastolic blood pressure; pSBP, peripheral systolic blood pressure; SEVR, subendocardial viability ratio; Total, means of main effect of time.

^a^ Condition main effect (*p* < 0.05).

^b^ Time main effect (*p* < 0.05).

Main effects of Condition were detected for AIx@75 ($\eta_{p}^{2}=0.45$), HR ($\eta_{p}^{2}=0.44$), and SEVR ($\eta_{p}^{2}=0.49$) (all *p* < 0.05), in which AIx@75 and HR were significantly higher and SEVR was significantly lower in ML compared to HL.

Main effects of Time were detected for AIx@75($\eta_{p}^{2}=0.34),$ cSBP($\eta_{p}^{2}=0.45)$, cDBP($\eta_{p}^{2}=0.43),$ pDBP($\eta_{p}^{2}=0.41)$, HR($\eta_{p}^{2}=0.32)$, and SEVR ($\eta_{p}^{2}=0.52$). AIx@75, cSBP, and HR significantly increased from BLto Post. cDBP and pDBP significantly decreased from BL to Post, while SEVR significantly decreased throughout the recovery period (*p* < 0.05). There were no significant Condition by Time interactions, or main effects for pSBP and cMAP (all *p* > 0.05).

## 4 Discussion

This study compared acute changes in indices of AS and central hemodynamics between two RT protocols with a high (HL) or moderate (ML) loading intensity (4RM vs. 12RM), while keeping the intensity of effort, repetition duration, and rest interval constant. Contrary to our hypothesis, ML promoted significantly greater increases in AS and pulse wave reflection, and significantly reduced myocardial perfusion when compared to the HL condition. These findings suggest that an acute bout of RT performed to volitional failure using a moderate loading intensity of 12RM places a greater demand on the arterial and cardiovascular system as opposed to a RT protocol with a higher loading intensity of 4RM.

The results of the present study are in partial agreement with a previous study assessing acute AS responses following RT protocols performed at different loading intensities [18]. Similarly to the current study, where cfPWV increased 14% immediately post ML exercise, Nitzsche et al. [18] reported a 13.8% and an 8% increase in cfPWV immediately post RT consisting of three sets of 30 reps at 30% of 1RM load (i.e., low load) and three sets of 20 reps at 50% of 1RM load (i.e., moderate load), respectively. Likewise, the protocol classified by Nitzsche et al. [18] as the “high load” (i.e., three sets of 10 reps at 70% of 1RM) did not induce any significant changes in cfPWV, a finding that was also supported in the current study. Contrary to the present study in which all sets were performed to volitional failure in an attempt to standardize effort, intensity of effort was not controlled in the study by Nitzsche et al. [18] where participants performed a fixed number of 10 repetitions. It has been argued that prescribing an arbitrary number of reps at a relative load based on a percentage of the maximal load an individual can lift (i.e., % of 1RM) limits comparisons within and between RT studies, since individual differences in muscular endurance among the studied population are completely disregarded [14]. Indeed, research has reported a great variability in the number of repetitions that can be performed by different individuals at the same relative load [13], indicating a different intensity of effort among them at the same loading intensity. Considering that cardiovascular responses seem to rely on the effort exerted during RT [31], it could be difficult to say whether differences between conditions in Nitzsche et al. [18] can be attributed exclusively to the different loads used, since heterogenous levels of effort may have blunted the study's findings.

Results of the present study are in contrast to the upheld belief that high loading intensity increases AS. Of note, the ML exercise protocol promoted significantly greater increases in cfPWV, AIx@75, and HR than HL. Specifically, the ML significantly increased cfPWV from BL values at Post (from 6.4 ± 0.3m/s to 7.3 ± 0.4 m/s) and15Post (6.8 ± 0.4m/s). Regardless of load, be it high (>80% 1RM), moderate (60%–80% 1RM), or low (<60% 1RM), mixed effects on AS have been reported in the literature following acute RT schemes [4, 5, 18]. Of note, this is the first study investigating the independent effects of load on AS, while maintaining a set of RT variables (e.g., intensity of effort, repetition, and rest duration) constant between experimental conditions. Importantly, standardization of effort adopted in the current study allows for a more appropriate investigation of other RT variables (i.e., load in this instance). This notion is supported by previous meta‐analyses examining the effects of single RT variables from a strength/hypertrophy standpoint, as they have only included studies that performed to volitional failure as a means of controlling the intensity of effort [7, 32]. Since differences in AS and pulse wave reflection parameters between different loading intensities are not yet fully understood, some speculations can be made based on previous research examining cardiovascular responses between various training loads while having a set of RT standardized between conditions [11, 33]. Vale et al. [33] reported that RT at high intensity of effort (i.e., both conditions reps were performed to volitional failure) with lighter loads and higher number of repetitions resulted in greater sympathetic activation in hypertensive postmenopausal women compared to heavier load—lower repetition RT protocol. Similarly, Gjovaag et al. [11] reported that a 20RM RT session resulted in higher blood pressure in young healthy adults than a 4RM RT when performed to volitional failure. In accordance, results of the present study indicated a significant greater increase in HR in the ML condition, indicative of sympathetic activation, while cSBP and pSBP also increased to a greater degree in the ML condition, although these changes were not significantly different from the HL. Considering the strong associations between AS with blood pressure and sympathetic activity [34, 35], it can be argued that additional, other than load, RT variables such as volume and time under tension might be related to increased AS following RT [11].

Potential mechanisms that might also explain the differences in arterial responses between loading intensities include improved vascular function, through increased nitric oxide (NO) expression and endothelial progenitor cell (EPC) mobilization [36, 37]. Güzel et al. [36] reported that NO production was greater in healthy males following a high load (80%–95% 1RM) compared to a low load (20%–35% 1RM) RT session. Elsewhere, Ribeiro et al. [37] examined the impact of acute RT at several loading intensities (i.e., 60%, 70%, and 80% 1RM) on the mobilization of circulating EPCs, which may indicate improved endothelial function. The authors reported a dose‐response relationship, with the highest loading intensities promoting the highest increases in EPCs both immediately and 6 h after the RT session. Future research is needed to confirm if these findings translate into improvement in AS following RT and to solidify these hypotheses.

Data presented herein demonstrated that measures of pulse wave reflection were significantly affected by the RT protocols. Changes in AIx and AIx@75 were significantly greater in ML than HL. Specifically, AIx@75 significantly increased (from −5.1 ± 3.2 to 0.5 ± 7.6%) in ML and (from −5.7 ± 4.3 to −2.9 ± 5.8%) after the HL session. Such findings are consistent with previous research reporting increases in AIx@75 (from 4.4 ± 8.1 to 23.0 ± 11.7%) following acute RT [4]. Nonetheless, increases in AIx@75 have also been observed in time‐matched to RT sedentary controls [38] or following acute aerobic and HIIT training [39], despite being well‐acknowledged that aerobic and HITT interventions reduce AS [40]. Along this line, it can be speculated that such effects may represent a physiological stress response that may not necessarily translate to harmful long‐term adaptations. That assumed, despite raising blood pressure, it has been recently proposed that wave reflections attenuate increases in blood flow that may protect against flow pulsatility to the microcirculation [41]. Yet, this is still speculative so future studies are needed to validate this notion.

In the present study, cSBP significantly increased post‐exercise. These findings are in agreement with some [38, 42] but not all previous studies [4, 43]. In addition, the current data did not show any changes in pSBP in any time point for both conditions, supporting previous assumptions that cSBP responses occur independent of changes in pSBP and AS [44]. Of note, cDBP significantly decreased immediately post‐exercise. Exact causes of this decrease remain unclear, as it is usually shown that that cDBP remains unchanged up to 10 min after acute RT [4, 38], although significant decreases have also been reported [42]. A similar trend was observed for SEVR which significantly decreased after both conditions throughout recovery. Given that myocardial perfusion occurs mostly during diastole, it has been suggested that a reduction in cDBP may result in a decrease in coronary blood flow [45]. Although previous studies reporting decreases in SEVR values after acute RT [4, 38] have not observed any parallel reductions in cDBP. Furthermore, since the extent of myocardial perfusion is primarily determined by DPTI, which in turn is mediated by increases in HR, it should be noted that increases in HR post‐exercise were observed, and although significantly different from baseline, these increases were moderate with mean values reaching at 75.5 ± 16 bpm. Given the paucity of available data, future research is needed to elucidate potential relationships and to ascertain whether these transient changes in cDBP and SEVR may represent harmful long‐term implications. Nevertheless, data from the current study indicate that RT performed at higher loading intensities promote a more favorable effect on coronary hemodynamics, potentially representing a safer RT approach especially for populations at risk.

Some limitations of the current study warrant consideration. It should be noted that volume load (i.e., set × repetitions × weight lifted) of ML was twice that of HL. Although volume load could have been equated between conditions, lighter loads inherently result in greater volume load than heavier load RT protocols [46]. Thus, whilst scientifically valid, this approach would have limited ecological validity and transfer to real training settings. For the same reason, the Valsalva manoeuvre was not controlled in this study despite its association with AS [47], since use of it may be unavoidable and often beneficial when lifting heavy loads or performing repetitions close to momentary failure [48]. In addition, in the case of some participants the specific repetition tempo prescribed could not be adopted during the final repetitions. Nonetheless, repetition velocity is usually reduced as a set approaches the point of failure, indicating the interdependence of RT variables [32]. Lastly, the current sample consisted of both males and females, thus sex‐related differences in vascular responses to RT might be a confounding factor. Despite these limitations, the current study adequately controlled a set of RT variables (i.e., intensity of effort, rest duration) to investigate the independent effects of load on AS. In addition, the present study was structured as a within‐subjects design thus minimizing inter‐individual variability in physiological responses to RT [49].

## 5 Conclusion

The current study suggests that performing RT to volitional failure using a moderate loading intensity (12RM) promotes significantly greater increases in measures of AS and pulse wave reflection in healthy individuals when compared to a high loading intensity 4RM. Collectively, these data suggest that RT protocols with lower loads and higher repetitions impose a greater workload on the arterial and cardiovascular system in comparison to RT schemes with heavier loads and lower repetitions. Findings of the present study may have important implications for the prescription of RT programs particularly from a cardiovascular‐health point of view. Future research should examine the potential implication of additional RT variables, such as intensity of effort, under volume‐equated conditions, in an attempt to elucidate the independent and synergistic effects of various RT variables on AS.

## Author Contributions

Conceptualization: E.K.; Writing/original draft preparation: E.K.; Investigation/testing: E.K.; Data analysis: E.K., J.F., S.H.; Review and editing: J.F., S.H., H.R.‐S. All authors have read and approved the final version of the manuscript and agree with the order of presentation of the authors.

## Ethics Statement

The study was approved by the Faculty of Health and Wellbeing of the University of Winchester (HWB_REC_230811_Karanasios). Experimental procedures were conducted following the approved ethics submission document. All participants received written information explaining the procedures and purpose of the study and gave their written consent prior to data collection.

## Conflicts of Interest

The authors declare no conflicts of interest.

## Data Availability

The data collected and analyzed for this study are available from the corresponding author upon request.
